# Editorial: Emerging Areas in Extracranial Carotid Stenosis Evaluation and Management

**DOI:** 10.3389/fneur.2022.891883

**Published:** 2022-04-06

**Authors:** Hege Ihle-Hansen, Peter Kelly, Christopher Bladin, Seemant Chaturvedi

**Affiliations:** ^1^Stroke Unit, Department of Neurology, Oslo University Hospital, Oslo, Norway; ^2^HRB Stroke Clinical Trials Network Ireland, University College Dublin, Dublin, Ireland; ^3^Stroke Service, Mater Misericordiae University Hospital, Dublin, Ireland; ^4^Department of Neurosciences, Monash University, Melbourne, VIC, Australia; ^5^School of Medicine, University of Maryland, Baltimore, MD, United States

**Keywords:** extracranial carotid stenosis, atherosclerosis, ischemic stroke, secondary prevention, atheroinflammation, plaque, cognition

Extracranial internal carotid artery stenosis is a leading cause of ischemic stroke. Patients can reduce their risk of future stroke with treatment with intensive medical therapy. In addition, selected patients can benefit from revascularization with carotid endarterectomy (CEA) or carotid stenting. Imaging methods for carotid stenosis evaluation have evolved considerably since the original randomized trials evaluating CEA that started more than three decades ago. These developments offer the prospect for more refined individual decision making for patients with carotid stenosis.

Today, stroke physicians on call assess and identify internal carotid artery stenosis on duplex ultrasound or CT angiography as part of the acute diagnostic work up and decision making regarding the potential cause and most beneficial intervention in the acute phase (1, 2). Measures of vascular burden and atherosclerosis as a subclinical disease can be included in optimizing primary and secondary prevention of vascular disease. Evaluation of extracranial vessels represents an important strategy to guide treatment decision making to improve outcome after stroke.

In this Research Topic, the editors aimed to summarize selected advances in carotid stenosis, including medical and surgical treatments. In line with the rapid development of new diagnostic and therapeutic approaches in stroke treatment, we aimed to explore the different new approaches for evaluation and management of extracranial carotid stenosis. Ten different publications report on novel aspects of risk factors, treatment, inflammation and use of advanced imaging modalities for plaque and stenosis, and extra cranial carotid stenosis as a cause of stroke, in prediction of prognosis and relation to cognition.

In the paper “*In Asymptomatic Carotid Disease and Cognitive Impairment: What Is the Evidence?”* Baradaran et al. review the current evidence on the relation between different manifestations of carotid disease and cognitive dysfunction, requesting longitudinal studies and streamlined diagnostic criteria regarding cognitive impairment. Like Ihle-Hansen et al. in “*Subclinical Carotid Artery Atherosclerosis and Cognitive Function: A Mini-Review*,” they report a significant association of carotid atherosclerosis and cognitive decline, and propose screening of carotid artery atherosclerosis to identify people at increased risk of cognitive impairment and to guide optimal risk factor management. Nuotio et al. report an association between long-term warfarin anticoagulation with increased calcification of carotid atherosclerotic plaques in the paper “*Warfarin Treatment Is Linked to Increased Internal carotid Artery Calcification*.” In “*Vascular Diameters as Predictive Factors of Recanalization Surgery Outcomes in Internal Carotid Artery Occlusion*,” Yan et al. introduced a risk stratification model to predict success rate of revascularization surgery. Further, in “*Nonstenotic Carotid Plaques In Embolic Stroke of Unknown Source*,” Kamtchum-Tatuene et al. discuss current knowledge regarding the association between embolic stroke of undetermined source (ESUS) and ipsilateral non-stenotic carotid plaque. Evans et al. demonstrate an independent association between atheroinflammation within carotid atherosclerosis and the severity of small vessel disease in “*Carotid Atheroinflammation Is Associated With Cerebral Small Vessel Disease Severity*,” indicating a future anti-inflammatory therapeutic approach to reduce the burden of chronic small vessel disease. Giannotti et al. combined PET and MRI markers of inflammation and of plaque stability to assess plaque vulnerability in “*Association Between 18-FDG Positron Emission Tomography and MRI biomarkers of Plaque Vulnerability in Patients With Symptomatic Carotid Stenosis*.” In addition, Nies et al. propose the inclusion of MRI biomarkers to assess plaque vulnerability in prediction models for stroke recurrence in “*Emerging Role of Carotid MRI for Personalized Ischemic Stroke Risk Prediction in Patients With Carotid Artery Stenosis*.” Finally, Lui et al. presented an uncommon etiology of stroke in the young; “*Hyoid Elongation May Be a Rare Cause of Recurrent Stroke in Youth-A Case Report and Literature Review*” Liu et al.

Still, to be able to compare results from different studies and to further explore the effect of interventions and the potential for including measures of plaques and stenosis in prediction models, we need standardization of methods to assess, define and report pathologies. The medical community also needs modern randomized trials to compare revascularization vs. intensive medical therapy (3), including long-term follow-up. It would be ideal to include cognitive outcomes as part of these trials.

Through these publications, our contributors have moved our knowledge a further step forward. Characterizing the nature and severity of extracranial carotid stenosis as part of regular stroke care may lead to improvements in outcomes that are meaningful to both patients and clinicians.

## Author Contributions

Guest editors, HI-H drafted the first version of the editorial. All authors contributed and approved the final version.

## Conflict of Interest

The authors declare that the research was conducted in the absence of any commercial or financial relationships that could be construed as a potential conflict of interest.

## Publisher's Note

All claims expressed in this article are solely those of the authors and do not necessarily represent those of their affiliated organizations, or those of the publisher, the editors and the reviewers. Any product that may be evaluated in this article, or claim that may be made by its manufacturer, is not guaranteed or endorsed by the publisher.
